# MRI in Prostate Cancer

**DOI:** 10.5812/ircmj.16620

**Published:** 2013-12-05

**Authors:** Mahyar Ghafoori, Manijeh Alavi, Mounes Aliyari Ghasabeh

**Affiliations:** 1 Department of Radiology, Department of Radiology, Hazrat Rasoul Akram University Hospital, School of Medicine, Iran University of Medical Sciences, Advanced Diagnostic and Interventional Radiology Research Center, Tehran, IR Iran; 2 Deputy of Research and Technology, Ministry of Health and Medical Education, Tehran, IR Iran; 3 Advanced Diagnostic and Interventional Radiology Research Center, Tehran University of Medical Sciences, Tehran, IR Iran

**Keywords:** Prostate cancer, Magnetic Resonance Imaging, Magnetic resonance Spectroscopy

## Abstract

Imaging studies play an important role in detection and management of prostate cancer and MRI especially with the use of endorectal coil because of high contrast resolution is recognized as the best imaging modality in evaluation of prostate cancer. Multiparametric MR study including T1 and T2 weighted images, diffusion weighted images, dynamic contrast study and MR spectroscopy is useful for detection and local staging of prostate cancer as well as posts treatment evaluation of patients either after surgery or radiation therapy for detection of local recurrence.

## 1. Context

Prostate cancer is the second most frequently diagnosed cancer and the sixth leading cause of cancer death in males worldwide; it is least common in South and East Asia, more common in Europe, and most common in the United States (1). According to the American Cancer Society, Prostate cancer is the most common malignancy in American men and the second leading cause of deaths from cancer, after lung cancer (2). The estimated lifetime risk of being diagnosed with the disease in USA is 17.6% for Caucasians and 20.6% for African. Based on 2008-2010 data, 15.33% of men born today worldwide will be diagnosed with prostate cancer some time during their lifetime. Therefore prostate cancer is likely to complicate the lives of a significant proportion of men that are healthy today (3, 4).

Like other cancers, early detection of new onset tumor leads to early treatment and decrease in mortality rate. Screening men for prostate cancer are done by PSA and digital rectal examination. The purpose of the screening is to detect early, tiny, or even microscopic cancers that are confined to the prostate gland. Early detection and early treatment of prostate cancer can stop the growth, prevent the spread, may reduce chance of dying and possibly cure the cancer (5-7).

## 2. Evidence Acquisition

### 2.1. Imaging Studies in Prostate Cancer

Almost all imaging modalities play a role in prostate cancer detection and management. Here at first we introduce briefly the application of ultrasonography and CT scans in prostate cancer and then discuss in details about MRI.

### 2.2. Ultrasonography

There are two different methods for ultrasonography of prostate. The first one is suprapubic approach using low frequency transducers that is suitable for measuring size and volume of prostate gland but has low resolution for evaluation of prostate anatomy and searching for tumors.

The second method is endorectal approach using high frequency endocavitary transducer. Transrectal ultrasound (TRUS) and TRUS guided biopsy play a significant role in diagnosing of prostate cancer. At present TURS is commonly the first imaging modality used for the initial evaluation of men suspicion of prostate cancer (8). TURS is useful for determining prostate size, zonal anatomy and detection of prostate cancer. TRUS also provides guidance for radiotherapy, brachytherapy, cryoablation, and high-intensity focused ultrasound (HIFU) (9). Peripheral zone of prostate is normally hyperecho in TRUS but prostate cancer is usually a hypoecho lesion within the peripheral zone. TURS are limited by high false-negative rates because 37% to 50% of malignant lesions in peripheral zone are isoechoic or only slightly hypoechoic and are not recognized by TURS. TURS also have limited ability to detect central gland malignancies (10-12).

Improvements of ultrasound technique in recent years lead to increase accuracy of TURS in detecting prostate cancers.

Doppler Ultrasound detects vascularity in a tissue and because cancerous tissue have always increase micro vasculature, Doppler Ultrasound can detect cancerous region in prostate, however in benign prostate hypertrophy and prostatitis also vascularity increases and Doppler Ultrasound only 5% to 17% increases the rate of prostate cancer detection over conventional gray scale (13, 14).

Contrast-enhanced US can detect micro-neovascularization and even is sensitive to low blood flow (Conventional color/power Doppler US cannot detect micro vascularization). Although contrast-enhanced US shows more sensitivity than conventional TURS in evaluating prostate cancer but performing it for detection of prostate cancer remains investigational (15, 16).

### 2.3. Computed Tomography (CT Scan)

CT scan is not used for evaluation of prostate gland itself because of low contrast resolution but can be performed for evaluation of pelvic lymphadenopathy in high risk men with prostate cancer but because of low sensitivity (35%) this method is not recommended (17).

Sclerotic skeletal metastasis can also be detected by CT scan.

### 2.4. Magnetic Resonance Imaging

Prostate MRI provides more clear and detailed images of the soft-tissue structures of the prostate gland than other imaging methods. The level of details and high resolution of images makes MRI an invaluable tool in early diagnosis and evaluation of prostate cancer (18). In order to intensify the signals and improve the clarity of MR images several variations of MRI coils are used. There are two kinds of coils that are used for prostate cancer imaging: 1- torso or pelvic (body) phased-array coil that is used on the surface of body. 2- Endorectal coil that is inserted into the rectum.

For imaging of prostate gland we can use only pelvic coil, only endorectal coil or combination of endorectal and pelvic coils. Although endorectal coil establishes higher image quality than pelvic coil due to the proximity of the coil to the prostate and higher signal to noise ratio but the accuracy rate for prostate cancer detection and staging range widely from 51% to 89% with endorectal coil alone. The best image quality achieves with using combination of endorectal and pelvic phased-array coils (19, 20).

Simultaneous using of endorectal and pelvic coils can best help in prostate cancer detection and evaluation of tumor extension.

Another important point that influences prostate imaging is strength of MR machines magnet. Magnetic field of MR scanners works based on two variables: uniformity of field density and strength of magnet. Commonly clinical MR scanners have 1.5 tesla (T) magnet strength. 3.0 T MR machines are also available and can be used for prostate cancer imaging. The clinical efficacy of these two MR scanners was compared in diagnosing disease and improving disease management (21). 3-T scanner shows faster and better performance than 1.5 T scanners. MR scanners are used in different clinical area and according to these area 1.5 T and 3-T scanners are showed different performance; for example in cerebrovascular imaging, coronary artery imaging and renal artery stenosis 3-T scanner provide higher image quality than 1.5T but in pelvic imaging and prostate cancer detection 1.5T scanners are better than 3-T scanners (22).

In prostate imaging two property of MRI are important: 1. Strength of MRI magnet; 1.5 T or 3-T 2. Type of MRI coil use for imaging; endorectal coil or torso/pelvic coil or combination of these two coils.

Standard 1.5 T scanners have capability of using both endorectal and pelvic coils and to get high resolution images, staging of prostate cancer and detection of tumor spreading around the pelvis these scanners are usually preferred.

3-T scanners with a higher filed strength although provide faster imaging sequences and higher imaging resolution than 1.5 T scanners but because of that high field strength we are not able to use commercially available endorectal coils with 3-T scanners yet and only the surface pelvic coil is currently available for 3 T MR machines.

Some studies also describe that because of high echo-train length used in 3-T MRI which causes motion or blurring, the rate of artifacts in images increases (23).

Different studies support that using 1.5 T MRI with combination of endorectal and body surface coil in prostate imaging give us images with higher quality than 3-T MRI with only pelvic coil (24-27), although it is supposed that generation of endorectal coil for 3-T MR machines will improve prostate cancer detection rate in the future (28).

According to above statements it can be concluded that the gold standard approach for diagnosis, staging and management of prostate cancer is using 1.5 T MR machines with both endorectal and pelvic phased-array coils.

## 3. Results

### 3.1. Detection of Prostate Cancer

**Figure 1.**
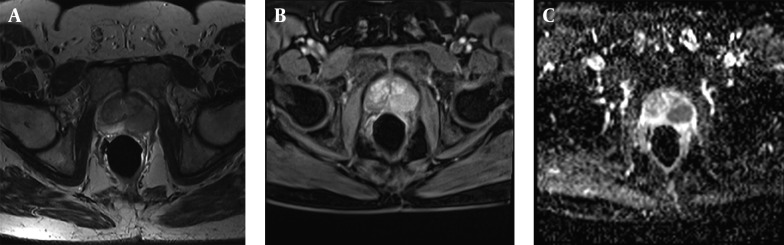
Biopsy Proven Prostate Cancer, Stage T2, Gleason Score 7. A) Axial T2w. image of prostate gland: A hyposignal tumoral mass is well depicted within the peripheral zone at the left side of prostate. Endorectal coil is seen in the lumen of rectum. B) Post contrast fat suppressed T1w. image: tumoral mass shows enhancement after contrast injection. C) Diffusion weighted image: water restriction is noted in tumoral lesion in ADC map.

High quality MR images display prostate zonal anatomy perfectly and pathologies of prostate as well. T1-weighted images illustrate prostate as homogeneous low signal intensity organ in which zonal anatomy and prostate cancer distinguishing is very difficult. T1-weighted is a useful image in detecting Post biopsy hemorrhage in prostate cancer (appears as high T1 signal intensity areas within the homogeneous prostate) and assess lymph nodes and osseous structures in pelvis (29-30). T2-weighted high resolution images are the mainstay in prostate cancer detection. The normal peripheral zone of prostate has high signal intensity in T2-weighted images. 70% of prostate cancers occur in peripheral zone and manifest as low signal intensity areas within the bright peripheral zone on T2-weighted MR images (Figure 1). Although T2-weighted MR images are useful in detecting prostate cancer but many pathologic features like post biopsy hemorrhage, hormone or radiation therapy effects, prostatitis, calcification and fibrosis appear as low signal intensity lesions in peripheral zone and mimic prostate cancer (29). In addition, heterogeneity in central glands signal intensity especially in patients with benign prostate hyperplasia overlap with tumoral tissue signal and makes detection of central glands cancer difficult. To overcome these limitations and to improve diagnostic accuracy of conventional MRI in prostate cancer detection, new techniques titled functional MRI have been developed. Functional MRI techniques include diffusion-weighted magnetic resonance (DW-MR) imaging, dynamic contrast-enhanced MR (DCE-MR) imaging and MR spectroscopy (30). 

### 3.2. MR Spectroscopy

**Figure 2.**
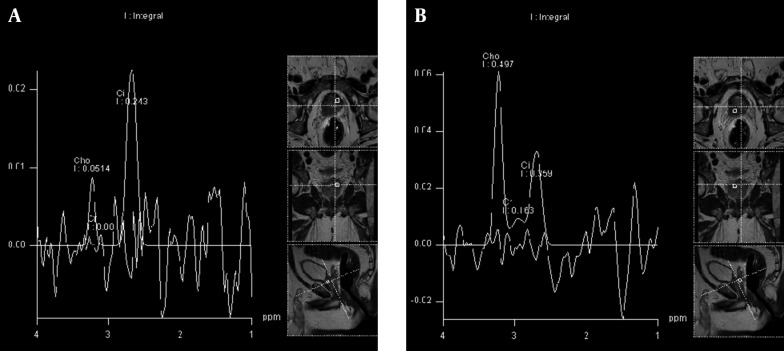
Magnetic Resonance Spectroscopy (MRS). A) MRS in non-tumoral portion of prostate gland: the choline peak is in low level and citrate peak is higher than choline that is considered as normal pattern. B) MRS from tumoral mass: Rise in choline peak and decrease in citrate peak have resulted in increase in choline to citrate ratio that is in favor of prostate cancer.

Magnetic resonance spectroscopy (MRS) is an enhance option to current MRI systems. MRS measures the level of specific metabolites in the prostate gland. These metabolites include choline (Cho) which is a metabolite of cellular turnover and its concentration increases in malignant tissue because of neoplastic cell production. Creatine (Cr) is another metabolite with spectroscopic peak very close to Choline and most of the times these two peaks are difficult to differentiate, then combination of choline and creatine is measured in MRS. The other metabolite that MRS measures is citrate (Cit). Cit tends to accumulate in peripheral zone and its concentration is high in normal prostate tissue but decreases in malignant tissues. MRS demonstrates these metabolites concentration as a spectrum. The ratio of Cho+Cr/Ci is used for evaluation of prostate cancer. Higher ratio is in favor of higher risk of malignancy. The ratio more than 0.75 is considered as significant and is consistent with prostate cancer (Figure 2) (31, 32). 

MRS not only helps in diagnosis of prostate cancer in peripheral zone but also is very useful in detecting cancer in transitional zone, anterior peripheral zone and apex of prostate (areas with difficult biopsy accessibility) when combine with T2 w. MRI, although prostatitis and post biopsy changes may interfere with MRS spectrum measurements (33).

MRS commonly perform after T2 w. MRI and different studies confirm that combination of MRS and MRI leads to better estimation of tumor aggressiveness and tumor staging, better tumor volume estimation and tumor localization (34) and also this combination is good in detection of disease recurrence after treatment (35, 36).

Recent studies showed that MRS is more accurate in detecting prostate cancers with high grade of malignancy and in low grade cancers its accuracy is somehow limited (37, 38).

### 3.3. Dynamic Contrast Study

Dynamic contrast enhanced (DCE) MRI works based on neo angiogenesis in tumor cells of prostate cancer. In DCE MRI, gadolinium contrast agent is injected intravenously by automatic power injector followed by flash of saline and then serial 3D T1- weighted images are obtained in multiple phases before, during and also after injection of contrast agent (39-42). In prostate cancer, like other cancers, angiogenesis rate is high and newly made vessels have low integrity in their wall thus they are more permeable than normal vessels. Fast leakage of contrast agent from leaky tumoral vasculature causes early enhancement of tumoral tissue in T1 - weighted MRI and also early wash out of contrast agent are seen in prostate cancer but these features are not shown in benign hyperplasia of prostate cell. To detect early enhancement of tumoral tissue and take reliable images; DCE MR is performed just before contrast injection up to 5 to 10 minutes after the injection. The 3D T1- weighted MRI set to take image every 5 to 10 seconds (Figure 2) (42, 43). 

Sensitivity and specificity of DCE MR in detecting prostate cancer have been calculated about 46–96% and 74–96%, respectively. DCE MRI shows better performance in tumors with more than 5 mm diameter (44, 45).

### 3.4. Diffusion Weighted Imaging

Diffusion Weighted Imaging (DWI) is a MRI method which works based on water molecules movements. Water molecules movement decrease in a high cellular environment and so diffusion become lower. Apparent diffusion coefficient (ADC) is the value which describes relation between cellularity and water diffusion and it has a revers relation with tissue cellularity. In prostate cancer because of cellules number increasing, water diffusion has restricted and ADC values are reduced (46, 47). DWI is a new technic and was initially applied to evaluate brain structures (48, 49) but improvement in MRI method and access to ultra-fast echo-planar sequences, gave us ability to apply DWI to investigate pelvic pathologies (50, 51). Sensitivity and specificity of DWI when added to T2-Weighted MRI for detecting prostate cancer is about 84% and 87% respectively; while T2-Weighted MRI alone has 65% sensitivity and 77% specificity (Figure 1) (52). 

### 3.5. Local Staging of Prostate Cancer

**Figure 3.**
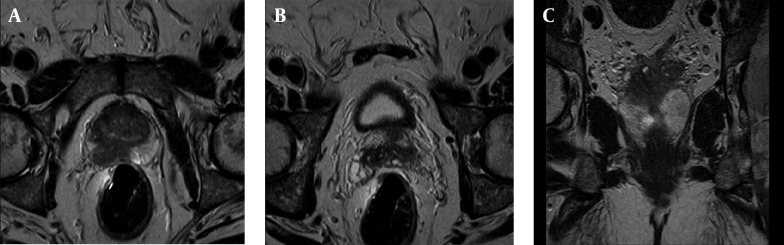
Biopsy Proven Prostate Cancer, Stage T3, Gleason Score 8. A)Axial T2w. image of prostate gland: A hyposignal tumoral mass is evident in peripheral zone in right base of prostate. B) Axial T2w. image of prostate gland in more cephalic level: Involvement of seminal vesicles by prostate cancer is noted. C) Coronal T2w. image of prostate gland: Involvement of seminal vesicles is well shown.

**Figure 4.**
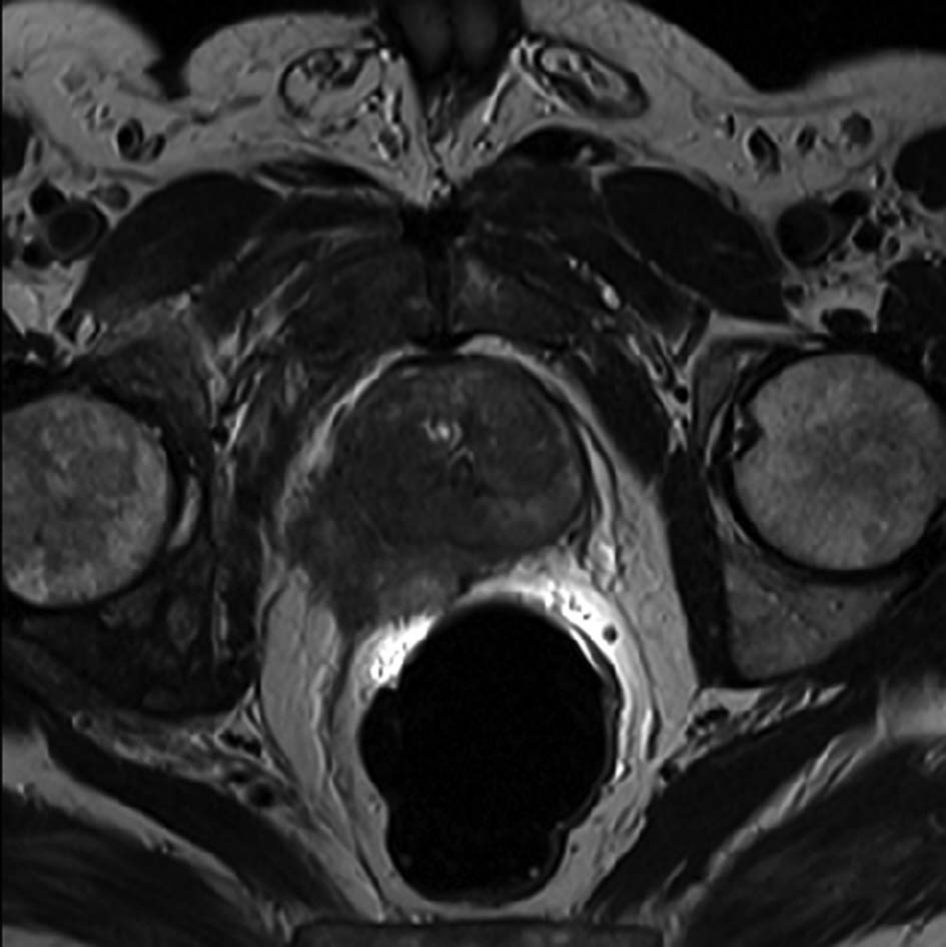
Axial T2w. image of prostate gland: A hyposignal tumoral mass is noted at the right side of prostate. The capsule of prostate is disrupted with invasion of tumor to periprostatic fat, neurovascular bundle and Denonvillier’s fascia.

Determining the extension of prostate cancer and local staging is one of the main roles of a radiologist after detection of prostate cancer. Staging of prostate cancer is very important in therapy decision making as well as prognosis determination. Imaging techniques play a significant role in staging of prostate cancer and MRI is the most accurate imaging modality used for prostate cancer staging (53). High resolution MR images especially with the use of endorectal coil can show with high accuracy whether the tumor is confined to prostate gland or there is involvement of prostate capsule and extra capsular extension that is considered the T component of TNM staging system. Extension of tumor to periprostatic fat, invasion to neurovascular bundles and involvement of seminal vesicles and Denonvillier’s fascia are well detected by MRI (Figures 3 and 4). 

Sensitivity and specificity of MRI for T staging is about 22%-75% and 73%- 99% respectively (26, 54). Adding dynamic contrast enhanced MRI to T2-Weighted MR images improves the accuracy for detection of extra capsular extension and seminal vesicle involvement and increases the sensitivity and specificity of local staging overall to 75% and 95% respectively (55). The point that should be considered is that MRI is usually used for local staging of prostate cancer in intermediate and high risk patient groups but it is useful in low risk patients as well.

Detection of lymph node involvement is the N component of TNM staging system and is well determined by MRI. Lymph node involvement is important in estimate of prostate cancer prognosis and disease recurrence.

Conventional MRI are only able to diagnose metastatic lymph nodes bigger than 10 mm but a newly invented MRI technique (lymphotropic superparamagnetic nanoparticles) detect occult lymph node metastasis smaller than 10 mm and has 100% sensitivity and 95.7% specificity in N staging (56).

Metastasis detection (M component of TNM staging system) is also important in choosing treatment and determining prognosis in prostate cancer. Bone is the most common sight for prostate cancer metastasis. MRI is useful for detection of prostate cancer metastasis in skeletal system as well as other body organs.

### 3.6. MRI Ability to Detection Bony Metastasis of Prostate Cancer

The most common site of the prostate cancer metastasis is axial bone and 80- 84% of all prostate cancer metastasis are located in axial bones (57). Bone scan is the first choice in detecting skeletal metastasis in a suspected patients beside radiologic studies but the false positive rate, especially after treatment, is high however although using bone scan in detecting bony metastasis of prostate cancer is steel unavoidable but we need more specific modality.

MRI is the most sensitive and specific technique in detecting bony metastasis. It is the best method in detecting bone marrow involvement in malignant disease (58, 59).

Evaluating prostate bone metastases is done best by MRI because MRI is more sensitive than other diagnostic techniques to early changes of metastatic bony tissues (60). Lecouvet et al. (58) in a study showed 100% sensitivity and 88% specificity (61) for bony metastasis detection by MRI (62). One of the limitations of MRI in bony metastasis detection was the lack of ability of whole body imaging which is solved by producing whole body coils although because of long lasting time of whole body MRI, it has limitation too (63). Unenhanced T1-weighted MRI added to turbo-short tau inversion recovery (STIR) sequences is highly sensitive to fat concentration changes of metastatic bone marrow. In metastatic bone marrow, increasing malignant cells leads to decrease water diffusion in tissue which is detectable by DW-MRI (64).

The most newly MRI technique which came to clinical practice is Whole-body DW imaging and it is very attractive because performing Whole-body DW imaging doesn’t need any radiation exposure or contrast agent injection and it takes a reasonable time. In Whole-body DW imaging, regular MR images and DW images are obtained from whole body of the patient simultaneously and then resulted images from both studies are composed and final image is overlap of high resolution MR images with highly sensitive DW images (like PET-CT or PET MR). Whole-body DW imaging is very helpful in detection of prostate cancer and its metastasis as well as post cancer therapy fallow up. Whole-body DW imaging has near 100% sensitivity is metastatic bone marrow lesion detection (65).

The potential role of Whole-body DW imaging in clinical practice is steel investigating (66, 67).

### 3.7. MRI in Targeting Biopsies

TRUS guided biopsy is the most commonly used method for prostate cancer detection in patients with high PSA level and/or abnormal DRE. Although TRUS guided biopsy reveals even clinically insignificant cancerous foci within the prostate but it is also probability to miss really malignant prostate cancer. TRUS has 35% false NPV (68). Different studies were shown that performing Multi parametric MRI of prostate and then using it as a guide for prostate biopsy either directly in MR machine or after overlapping the images on real time TRUS images with fusion imaging methods will increase the detection rate of prostate cancer and is especially important in patients highly suspicious of prostate cancer due to abnormal PSA level and previous negative biopsy result (69, 70).

### 3.8. Treatment Planning for Radiation Therapy

Radiation therapy is one of the main prostate cancer treatment methods that can be used in low stage up to high stage of disease and even in bone metastasis of prostate cancer. According to patients’ condition and stage of disease, radiation therapy performs alone or in combination with other therapy methods like surgery or hormonal therapy.

There are two kinds of radiation therapy methods: External beam radiation therapy (send X-ray or proton radiation to cancer from a machine outside the body) and Brachytherapy (implanting about 100 radioactive seeds in the prostate). Radiation therapy can use in low risk patients for curing the cancer completely and it calls radical radiotherapy. In higher stage of prostate cancer and when bone metastasis are existence and patients suffer from pain; radiation therapy is used for shrinking cancerous cells on nerves systems and bones to decrease pain (palliative therapy) (71). After radical prostatectomy radiation therapy is useful for preventing and decreasing cancer recurrence and distal metastasis. In intermediate to high risk patients, radiation therapy can be used in combination with hormonal therapy before or after radical prostatectomy (72). MR images can be used reliably for treatment planning of radiation therapy.

### 3.9. Evaluation of Local Recurrence After Treatment

**Figure 5.**
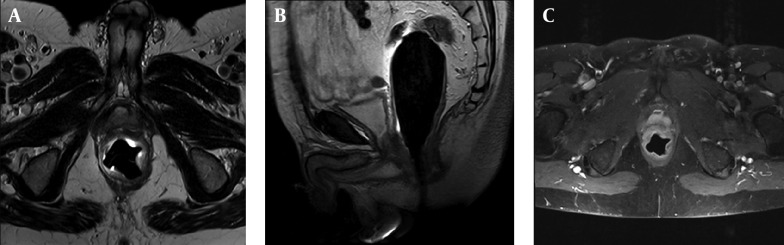
Patient with History of Radical Prostatectomy due to Prostate Cancer. The Patient was Suspicious of Tumor Recurrence Because of Gradual Rise in PSA. A) Axial T2w. image: prostate gland is surgically removed. A mass lesion is evident in posterior wall of bladder neck immediately above the anastomotic site that is suggestive of local recurrence of prostate cancer. B) Sagittal T2w. image: tumoral mass is well depicted in posterior wall of bladder neck. Endorectal coil is well seen in rectum. Note high signal intensity near to coil. C) Post contrast fat suppressed T1w. image: tumoral mass shows enhancement after contrast injection.

30% of patients underwent radical prostatectomy show recurrence of cancer. Vesicourethral anastomosis and retrovesical space are the most commonly sites for recurrence (Figure 5) (73, 74). Different studies were shown that conventional MRI has 48 to 100% sensitivity and 52 to 100% specificity in prostate cancer recurrence detection (75, 76). MR spectroscopy detects recurrence after radical prostatectomy with 84% and 88% sensitivity and specificity respectively. DCE-MRI has 71% and 91% sensitivity and specificity. Combination of MRS and DCE-MRI show 87% and 94% sensitivity and specificity respectively (77). DW-MRI is also capable to detect cancer recurrence after radical prostatectomy in patients that conventional MRI has missed recurrence (78). 

Prostate cancer recurrence may also happen after radiation therapy (0% to 80%) (79). The most common place for prostate cancer recurrence after radiation therapy is primary cancer site. DCE-MRI has 95% specificity and 68% sensitivity in cancer recurrence detection. DW-MR imaging alone shows low sensitivity in cancer recurrence detection after radiotherapy (25%) but in combination with T2-Weighted MRI, sensitivity increases to 62%. Specificity in both condition is acceptable (92% vs 97%).

## 4. Conclusion

High resolution Multiparametric MR imaging including regular T1 weighted and T2 weighted images accompanied by dynamic contrast-enhanced MRI, diffusion weighted imaging and MR spectroscopy obtained in 1.5 T MR machines with simultaneous use of pelvic and endorectal coils is the best imaging modality in prostate cancer and is useful for detection and local staging of prostate cancer, follow-up of patients after radical prostatectomy or radiation therapy to detect local cancer recurrence, detection of skeletal metastasis and also targeting biopsies in patients highly suspicious of prostate cancer but with previous negative TRUS guided biopsies.
